# Supplementary material for: Selective nucleophilic α-C alkylation of phenols with alcohols via Ti=Cα intermediate on anatase TiO2 surface
Source: Nat Commun. 2023 Aug 2;14:4479. doi: 10.1038/s41467-023-40101-7 (PMC10397351; doi:10.1038/s41467-023-40101-7)
Supplement: Supplementary file 1 — Supplementary information [file 41467_2023_40101_MOESM1_ESM.pdf]

Supplementary Information

Selective Nucleophilic α-C Alkylation of Phenols with Alcohols via Ti=C_{α} Intermediate at Oxygen Vacancy on Anatase TiO_2 Surface

Xinze Du,[a,c] Hongjun Fan,[b]* Shenglin Liu,[a] and Z. Conrad Zhang[a,d]*

[a] State Key Laboratory of Catalysis, Dalian National Laboratory for Clean Energy, Dalian Institute of Chemical Physics, Chinese Academy of Sciences, Dalian 116023, China

[b] State Key Laboratory of Molecular Reaction Dynamics, Dalian National Laboratory for Clean Energy, Dalian Institute of Chemical Physics, Chinese Academy of Sciences, Dalian 116023, China

[c] University of Chinese Academy of Sciences, Beijing 100049, China

[d] Changzhou University, Changzhou 213164, China

Table of contents

1. Conversion of primary alcohols in alkylation reactions **(Supplementary Table 1)** S3

2.Optimization of reaction conditions **(Supplementary Fig. 1)** S4

3. α-C alkylation reactions of substituted phenols and naphthols (**Supplementary Fig. 2**) S5

4. Reuse test of TiO_2-A **(Supplementary Fig. 3)** S6

5. XPS spectra of TiO_2-A **(Supplementary Fig. 4)** S7

6. Control experiments S8

7. Procedure of pretreatment of TiO_2-A to get Alc-TiO_2-A S9

8. NMR spectra of isotopic product **(Supplementary Fig. 5)** S10

9. Eyring study **(Supplementary Fig. 6)** S11

10. The distribution and diffusion of vacancies on TiO_2-A **(Supplementary Fig. 7)** S12

11. The mechanism of nucleophilic α-C alkylation on TiO_2-A **(Supplementary Fig. 8)** S13

1. Conversion of primary alcohols in alkylation reactions

Supplementary Table 1. Conversion of primary alcohol in alkylation of phenol. [a]

Entry	Catalysts	Alcohol	Conv. (%)	Alkylation product yield (%)	Alkyl phenyl ether yield (%)	Alkene yield (%)	Alkyl ether yield(%)
1	H-ZSM-5	1-propanol	67.2	16.9	1.9	27.4	4.8
2	γ-Al_2O_3	1-propanol	77.6	22.5	2.5	34.3	6.4
3	TiO_2-A	1-propanol	27.1	20.3	1.6	3.6	0.5
4	TiO_2-A	1-dodecanol	20.8	14.2	2.5	2.7	0.3
5	Alc-TiO_2-A	1-propanol	22.9	19.1	0.7	1.7	0.3

[a] Reaction conditions: catalyst 0.2 g, phenol 2.5 mmol, alcohol 10 mmol, toluene 25 mL, N_2 atmosphere, 300 °C, 16 h.

2. Optimization of reaction conditions

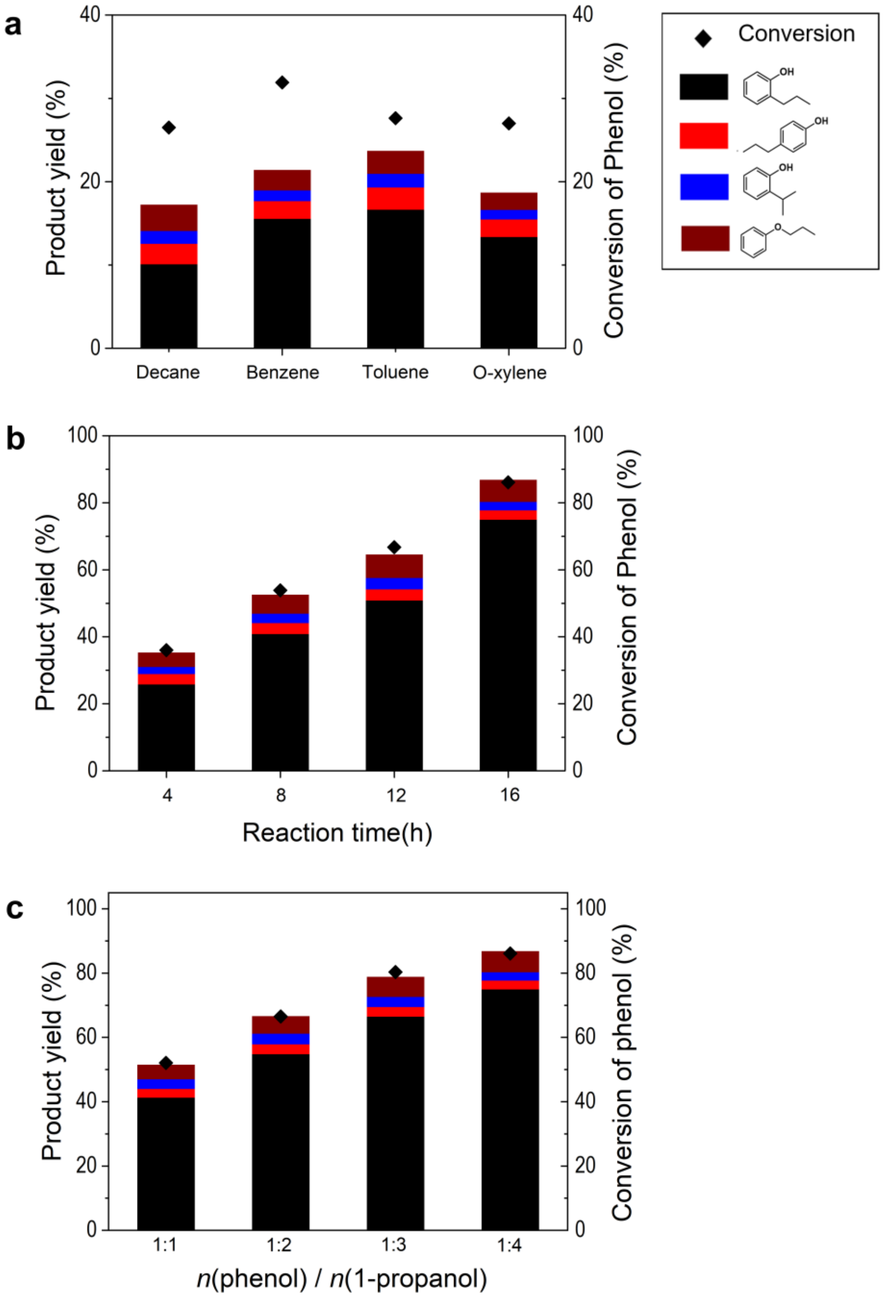

Supplementary Fig. 1 Product yields in the alkylation of phenol with 1-propanol. (a) effect of solvent; (b) effect of reaction time; (c) effect of feeding ratio between phenol and 1-propanol. Reaction conditions: TiO_2-A 0.2 g; phenol: 5 mmol in part (a), 2.5 mmol in part (b), as indicated in part (c); 1-propanol 10 mmol, solvent 25 mL, N_2 atmosphere, 300 °C; 4 h in part (a), as indicated in part (b), 12h in part (c).

3. α-C alkylation reactions of substituted phenols and naphthols

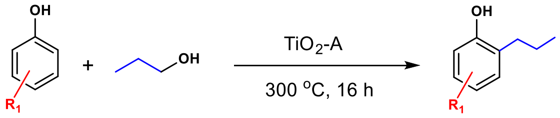

Entry	Phenol/ Naphthol	Conv. (%)	α-C Alkylation Product	Selectivity (%)
1	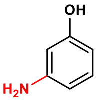	92.1	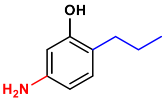	85.1
2	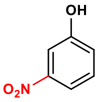	84.2	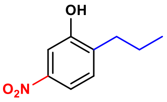	80.3
3	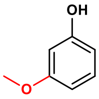	88.9	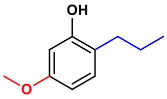	81.7
4	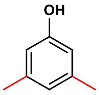	91.4	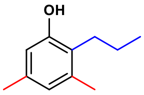	87.5
5	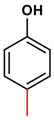	93.9	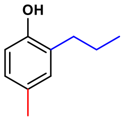	88.6
6	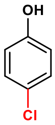	91.1	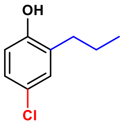	83.7
7	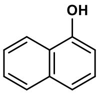	93.6	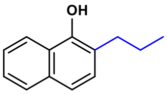	87.9
8	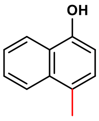	94.7	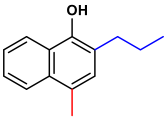	88.2
9	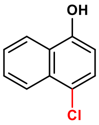	90.4	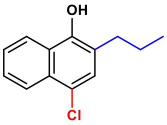	86.3
10	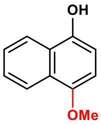	91.2	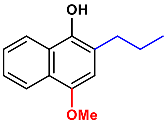	81.4

Reaction conditions: TiO_2-A 0.2 g, phenol or naphthol 2.5 mmol, alcohol 10 mmol, toluene 25 mL, 300 °C, 16 h, N_2.

Supplementary Fig. 2 The α-C alkylation of substituted phenols and naphthols with 1-propanol.

4. Reuse test of catalyst

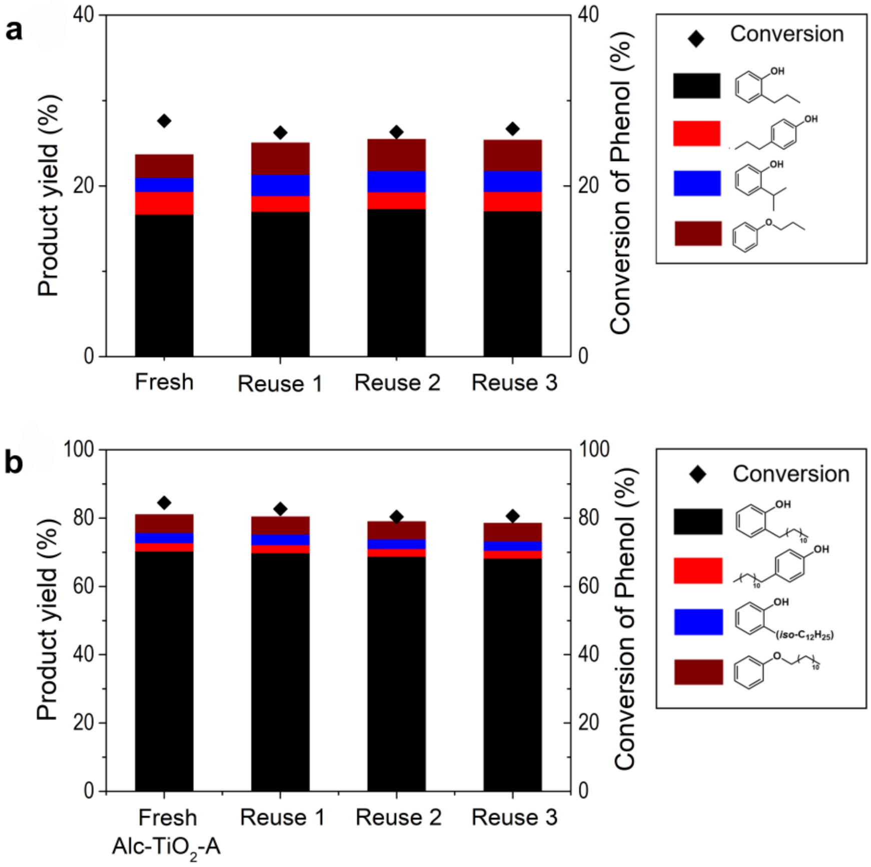

Supplementary Fig. 3 Reuse test of catalysts. (a) alkylation of phenol with 1-propanol on TiO_2-A; (b) alkylation of phenol with 1-dodecanol on Alc-TiO_2-A. Reaction conditions: catalyst 0.2 g, phenol 2.5 mmol, alcohol 10 mmol, toluene 25 mL, N_2, 300 °C, 4 h in (a) and 24 h in (b). The recycled catalyst was used without further pretreatment after separation from the reaction mixture in the previous test.

5. XPS spectra of TiO_2-A

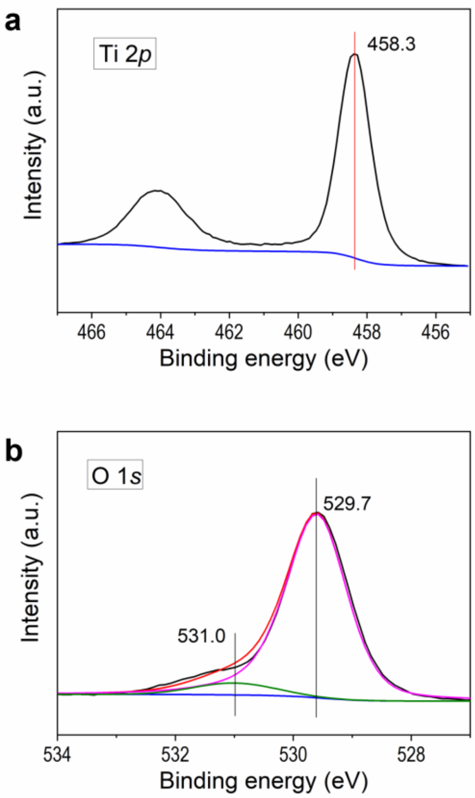

Supplementary Fig. 4 XPS spectra of TiO_2-A. (a) Ti 2*p*; (b) O 1*s*.

6. Control experiment

(1) Alkylation of phenol with alkenes.

Entry	Alkenes	Conversion(%)	*o*-n-alkylation product yield(%)
1	1-Hexene	1.0	0
2	1-Dodecene	1.4	0

Reaction conditions: TiO_2-A 0.2 g, phenol 5 mmol, alkene 10 mmol, toluene 25 mL, N_2, 300 °C, 4 h.

(2) Alkylation of phenol with n-propyl ether.

Entry	Conversion (%)	Product yield(%)			
		2-n-propylphenol	4-n-propylphenol	2-isopropyl phenol	n-propyl phenyl ether
1	8.9	4.7	1.6	2.2	0.6

Reaction conditions: TiO_2-A 0.2 g, phenol 5 mmol, n-propyl ether 5 mmol, toluene 25 mL, N_2, 300 °C, 16 h.

(3) Rearrangement reaction of n-propyl phenyl ether.

Entry	Conversion (%)	Product yield(%)			
		2-n-propylphenol	4-n-propylphenol	2-isopropyl phenol	Phenol
1	60.9	10.6	3.1	7.8	38.9

Reaction conditions: TiO_2-A 0.2 g, n-propyl phenyl ether 2.5 mmol, toluene 25 mL, N_2, 300 °C, 16 h.

7. Procedure of pretreatment of TiO_2-A to get Alc-TiO_2-A

Alc-TiO_2-A was prepared by treating TiO_2-A with 1-propanol under reaction conditions. In a typical run, 1 g TiO_2-A, 10 mmol of 1-propanol and 25 mL of toluene were loaded into the reactor. The reactor was purged with N_2 for 10 min to remove the air. The reactor was then heated to 300 °C and kept for a specified reaction time while the content was stirred at a rate of 500 rpm. After the reaction, the catalyst was washed with ethanol and separated by centrifugation. The sample was dried overnight at 110 °C and then heated to 400 °C at a rate of 10 °C/ min and kept at 400 °C for 2 hours in 10 vol% H_2/Ar and then cooled to room temperature under N_2 atmosphere to obtain Alc-TiO_2-A.

8. NMR spectra of isotopic product

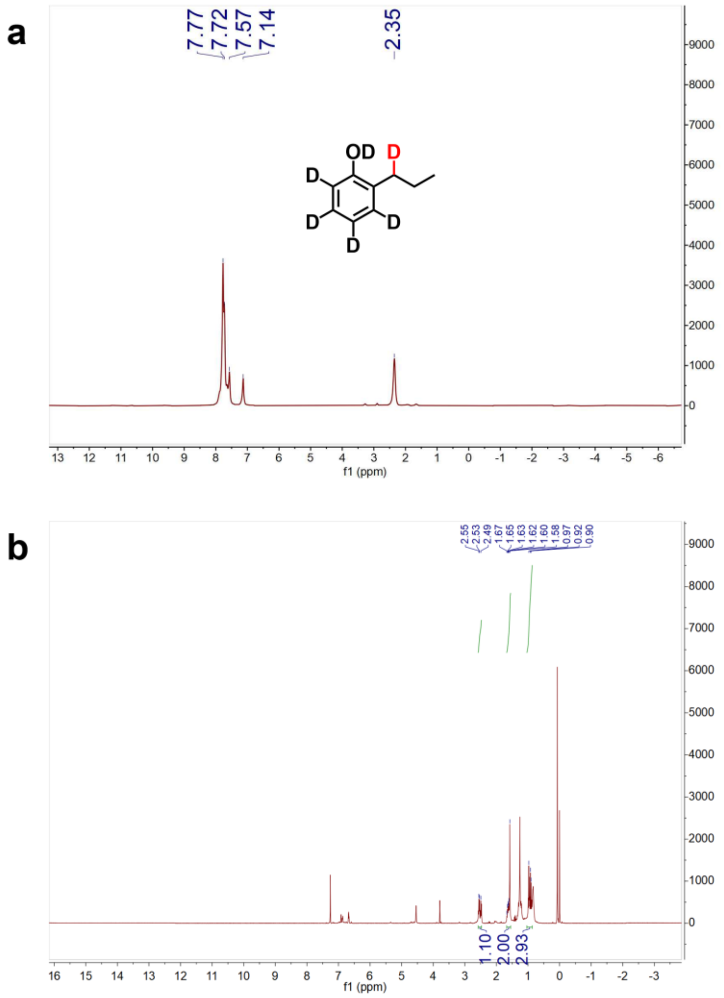

Supplementary Fig. 5 NMR spectra of isotopic product. (a) ^{2}H spectra; (b) ^{1}H spectra.

9. Eyring study

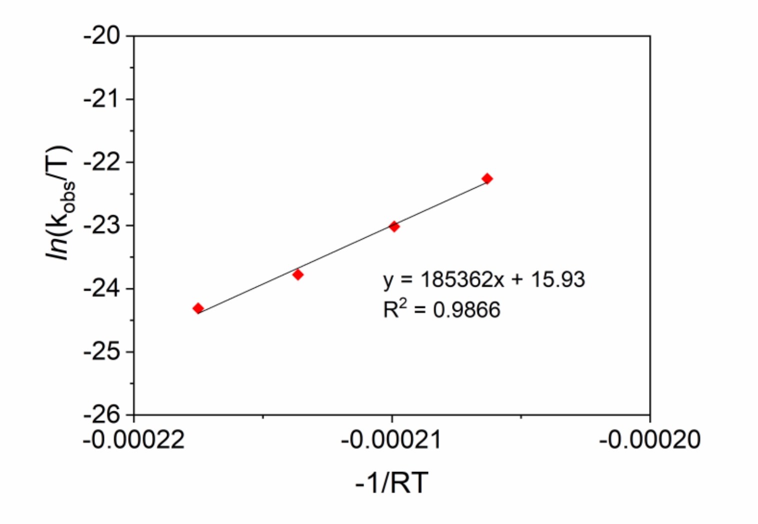

Supplementary Fig. 6 Eyring plot of *ln*(k_{obs}/T) vs. -1/RT.

The initial rate of the reaction was measured as a function of temperature (280, 290, 300, 310 °C) and Eyring analysis was performed to determine the activation parameters. The slope and intercept were used to calculate the activation enthalpy of the reaction $\Delta H^{\neq}$ = 185.4 kJ·mol^{-1} and the activation entropy $\Delta S^{\neq}$ = -65.1 J·mol^{-1}·K^{-1} as the Eyring Equation description:

$$ln\frac{k}{T} = \Delta H^{\neq} \times \frac{-1}{RT} + ln\frac{k_b}{h} + \frac{\Delta S^{\neq}}{R} \quad .$$

10. The distribution and diffusion of vacancies on TiO_2-A

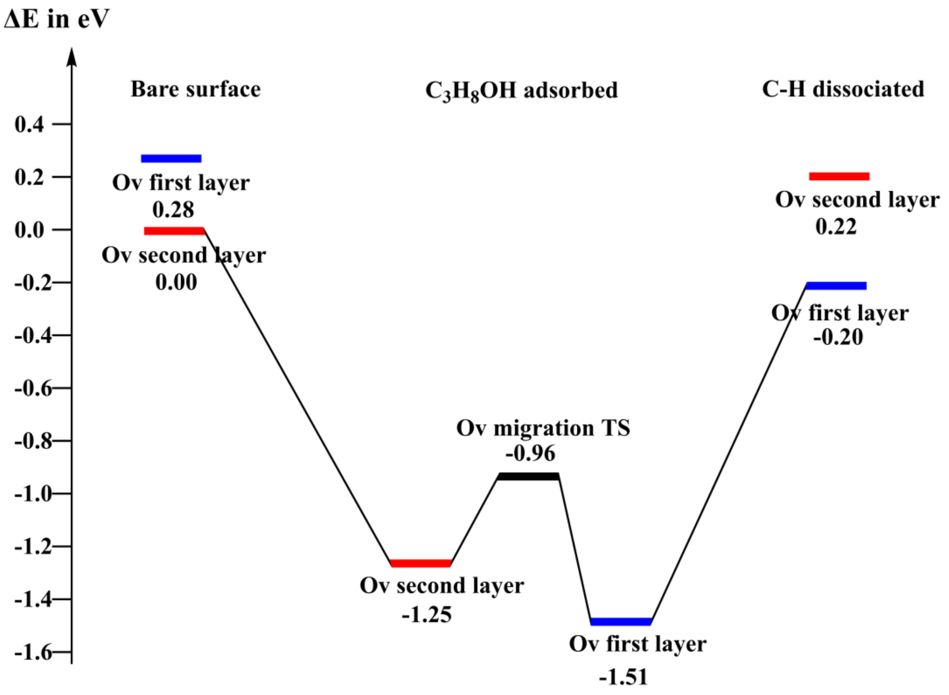

Supplementary Fig. 7 The distribution and diffusion of vacancies on TiO_2-A. Blue: oxygen vacancy on the first layer; Red: oxygen vacancy on the second layer.

11. The mechanism of nucleophilic α-C alkylation on TiO_2-A

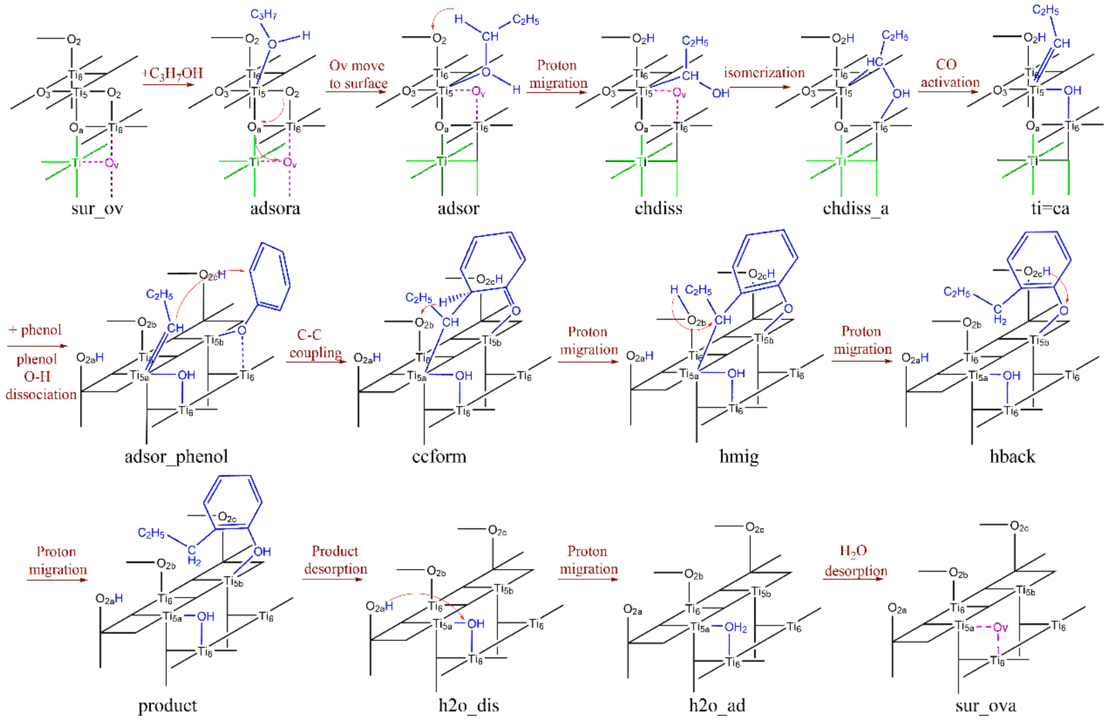

Supplementary Fig. 8 The mechanism of nucleophilic α-C alkylation on TiO_2-A.
